# The Anatomical and Evolutionary Impact of Pain, Pleasure, Motivation, and Cognition: Integrating Energy Metabolism and the Mind–Body BERN (Behavior, Exercise, Relaxation, and Nutrition) Framework

**DOI:** 10.3390/ijms26125491

**Published:** 2025-06-08

**Authors:** George B. Stefano, Pascal Buttiker, Maren M. Michaelsen, Tobias Esch

**Affiliations:** 1 Department of Psychiatry of the First Faculty of Medicine and General Teaching Hospital, First Faculty of Medicine, Charles University, 10000–10999 Prague, Czech Republic; gstefano@sunynri.org (G.B.S.); pascal_buettiker@hotmail.com (P.B.); 2 Institute for Integrative Health Care and Health Promotion, Witten/Herdecke University, 58455 Witten, Germany; maren.michaelsen@uni-wh.de

**Keywords:** mitochondria, evolution, mind–body medicine, BERN, motivation, CNS pathways and networks, hypoxia

## Abstract

In this manuscript, we highlight the evolutionary origins of mitochondria from bacterial endosymbionts and explore their contributions to health, energy metabolism, and neural–immune communication. Mitochondrial adaptability and the roles played by these organelles in promoting oxygen-dependent ATP production provide critical regulation of cognition, motivation, and inflammation. Hypoxia has been identified as an important initiator of inflammation, neurodegeneration, and mitochondrial dysfunction, emphasizing the overall importance of oxygen homeostasis to health and well-being. The Behavior, Exercise, Relaxation, and Nutrition framework highlights these observations as tools that can be used to optimize mitochondrial efficiency. Interestingly, mitochondrial dysfunction may also be linked to psychiatric disorders (e.g., schizophrenia), a hypothesis that focuses on energy dynamics, a proposal that may extend our understanding of these disorders beyond traditional neurotransmitter-focused concepts. Collectively, these perspectives underscore the critical contributions of mitochondria to health and disease and offer a novel framework that may help to explain the connections featured in mind–body medicine.

## 1. Introduction

**Figure 1:**
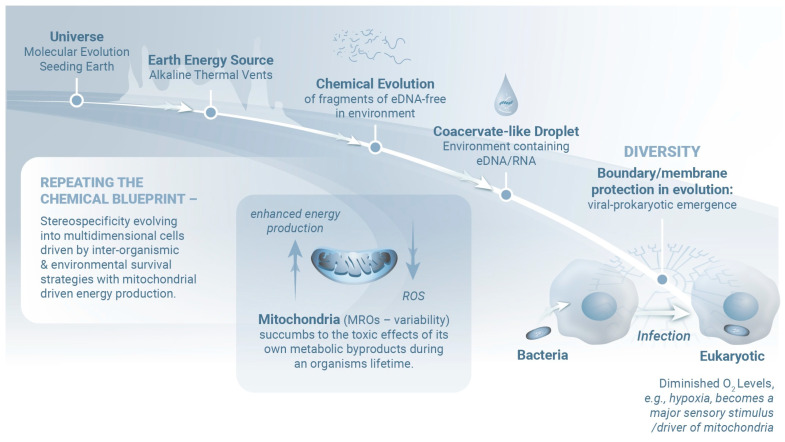
Evolutionary events that unfolded during the earliest stages of the Universe laid the foundations for life on Earth. Evidence of these events remains in the form of primordial molecules that have been detected on asteroids traveling in our solar system. Our current understanding suggests that the Earth maintained uniquely stable and hospitable thermodynamic conditions that facilitated further growth. Alkaline thermal vents provided the necessary energy needed by molecular fragments (for example, early nucleic acids) to combine and evolve. Environmental phase differences facilitated the formation of coacervate-like droplets containing RNA and environmental DNA (eDNA, i.e., extracellular DNA that can be detected in the environment), eventually giving rise to primitive membranes. Membrane development represented a critical advance, as these structures promote molecular stability and thus may facilitate efficient and rapid evolution, leading to the emergence of viral and prokaryotic life forms. Oxygen, a highly reactive and potentially toxic molecule, became a central focus after the development of organisms capable of photosynthesis. Ultimately, several bacterial species evolved the capacity to metabolize oxygen via pathways that simultaneously reduced the toxicity of this molecule and harnessed it for energy production. Endosymbiosis was a clear next step after this pivotal adaptation. Early pre-eukaryotic cells were “infected” and eventually incorporated/infected the oxygen-metabolizing bacteria that ultimately evolved to become mitochondria and mitochondria-related organelles (MROs), which are simpler versions of mitochondria found in eukaryotes living in low-oxygen environments. Over time, this relationship led to a refined version of intracellular communication. The mitochondria were tasked with oxygen detoxification, which at the same time provided energy and supported various metabolic functions in the host cell. Oxygen remains a fundamental regulator of mitochondrial activity. The evolutionary constraints in response and sensitivity to environmental pressures that led to the emergence of eukaryotic single cells eventually gave rise to multicellular organisms that were shaped by cell-to-cell interactions. Of note, although mitochondria are capable of counteracting oxygen toxicity, they eventually succumb to the long-term effects of their own metabolic byproducts. Over an organism’s lifetime, mitochondrial metabolic imbalance can disrupt energy homeostasis. This response gradually harks back to the initial infectious nature of mitochondria that was once obscured by their perceived symbiotic role. Modified from [19].

The presence of an evolutionarily conserved set of signaling molecules across diverse life forms strongly suggests the existence of a broad intermediate metabolic profile that relies on the high output of mitochondrial ATP [1]. Interestingly, the presence of salt minerals, amino acids, and all five DNA and RNA nucleobases on asteroid (101955) Bennu suggests that Earth may have been seeded with the essential building blocks for life, potentially supporting the planet’s biological evolution [2,3] (Figure 1). Established mechanisms of bidirectional neural–immune communication [4,5,6] observed in both invertebrate and vertebrate species require shared anatomical and biochemical substrates that contribute to both energy production and mitochondrial integrity. Within this interconnected network, tissue-specific disruptions can have profound and far-reaching effects on several associated systems (e.g., the central nervous system [CNS]). Given the need for a consistent oxygen supply, the primary trigger for physiologically dysfunctional responses might be a comparatively simple hypoxic event [1,7,8]. While brief fluctuations in oxygen levels can be tolerated, unresolved hypoxic events may initiate a cascade of self-amplifying pro-inflammatory responses [1] (Figure 1), notably, increased levels of circulating hypoxia-inducible factor (HIF), interleukin-1 receptor antagonist, IL-6, and C-reactive protein [9,10,11]. Interestingly, a state of excess oxygen, or hyperoxia, can also induce an inflammatory response [12].

Interestingly, hypoxia has emerged as a potentially effective treatment for neurodegeneration associated with Leigh’s syndrome, an inherited disorder resulting from deleterious mutations in the mitochondrial electron transport chain [13,14]. Jain et al. [15] reported that chronic hypoxia led to prolonged survival and improved locomotion of *Ndufs4*^-/-^ Leigh syndrome mice and that the biochemical HIF-prolyl hydroxylase inhibitor, FG-4592, stabilized HIF-1-mediated transcription in several antimycin- or oligomycin-treated human cell lines and genetically engineered zebrafish embryos maintained under normoxic conditions. Results from a subsequent study by Ferrari et al. [16] confirmed that chronic hypoxia prolonged the survival of *Ndufs4*^-/-^ mice and could reverse parenchymal inflammation and atrophy characteristic of late-stage disease. While the mechanisms underlying these pathways await future study, there is already some evidence linking Leigh syndrome to the pathogenesis of neuropsychiatric disease [17,18]. Although at first glance contradictory, this provides further evidence of the important and fine-tuned interplay between mitochondria and the cellular microenvironment under altered oxygen conditions, promoting oxygen homeostasis and reciprocal protection.

Mitochondria evolved from an ancestral bacterial species that established a symbiotic relationship with an early pre-eukaryotic cell [20,21,22] (Figure 1). This partnership likely persisted because the bacteria provided the eukaryotic cell with the capacity to detoxify oxygen generated by photosynthesis while boosting cellular energy production [21,22]. Although mitochondria were traditionally not considered “alive” because they were believed to be incapable of surviving independently, more recent findings reveal that mitochondria have their own DNA and can replicate autonomously within host cells. Functional mitochondria can also exist in the extracellular space [23], where they regulate cell-to-cell communication, tissue regeneration, and immune responses [24,25,26] and have been implicated in the pathogenesis of CNS disorders [27].

Mitochondria exhibit remarkable evolutionary plasticity, with the capacity to support anaerobic metabolism via transformation into hydrogenosomes, mitosomes, and intermediate forms collectively known as mitochondrion-related organelles [20]. These findings underscore the multifaceted roles played by mitochondria in both energy metabolism and as a force promoting the complexity of eukaryotic life [21,28]. Mitochondrial adaptability to diverse environments and the selective pressures that they impose have been pivotal in the origin and ongoing maintenance of eukaryotic cell complexity [28].

Mitochondria are uniquely positioned at the intersection of bioenergetics, redox signaling, and neuroimmune regulation. Thus, a specific focus on mitochondrial biology and function will provide mechanistic insight into the pathophysiology of cognitive and psychiatric disorders beyond what is offered if one maintains a singular focus on protein synthesis or membrane transport. In contrast to these processes, which are frequently downstream or compartmentalized, mitochondrial function directly governs neuronal excitability, neurotransmitter synthesis, synaptic plasticity, and stress responsivity by tightly coupling ATP production, calcium buffering, and generation of reactive oxygen species (ROSs) to meet cellular demands [29,30]. Mitochondria are especially critical in the brain due to the high and fluctuating energy demands of synaptic transmission and plasticity, processes that are foundational to learning, memory, and emotional regulation [31]. Mitochondria also modulate neuroinflammation through redox-sensitive pathways and the release of damage-associated molecular patterns. For example, mitochondrial DNA released from cells can activate innate immune sensors such as the NLRP3 inflammasome, providing a direct link between mitochondrial distress and psychiatric symptoms [32,33]. Furthermore, psychiatric disorders (e.g., depression, bipolar disorder, and schizophrenia) are increasingly associated with altered mitochondrial dynamics, impaired oxidative phosphorylation, and mitochondria–nuclear communication imbalances—features that are not readily explained by disruptions in protein synthesis or membrane function alone [31]. Moreover, recent evidence suggests that mitochondrial-targeted interventions (e.g., NAD^+^ boosters, antioxidants, and lifestyle factors/activators of mitochondrial biogenesis) can modulate both mood and cognitive outcomes [34,35]. Therefore, mitochondrial biology offers a multi-scale, integrative framework for understanding how stress, metabolism, and immunity converge to promote brain dysfunction.

Here, we begin by reviewing earlier findings on the impact of biological stresses (notably, hypoxia and inflammation) on mitochondrial function and the overall efficiency of energy production. We then go on to consider the contributions of these phenomena to our current understanding of the mechanisms underlying mind–body medicine and the pathogenesis of neuropsychiatric disease.

## 2. Molecular Mind–Body Platform

Behaviorally mediated practices eliciting the relaxation response [36] likely function effectively because they support the synchronization of the peripheral nervous system and the CNS [7,8]. The results of several studies suggest that controlled breathing exercises may promote reductions in respiratory rate, heart rate variability, and blood pressure [37,38,39]. Taking this one step further, we hypothesize that the neurocognitive pathways associated with pain, pleasure, and motivation may rely on the synchronization of energy metabolism based on a shared dependency on common networks and that synchronized energy metabolism is likely critical for the effective functioning of neurocognitive pathways associated with pain and pleasure (and likely others). While pain and pleasure pathways are linked mechanistically by shared neurotransmitter signaling (as reviewed in [40]), the CNS as a whole is critically dependent on mitochondrial function in neurons as the main source of ATP [41]. ATP is needed to maintain membrane potentials, synthesize neurotransmitters, and regulate synaptic activity [31]. We hypothesize that the mitochondrial activity must be synchronized across these shared circuits to ensure a balanced energy supply [42]. Disruptions in mitochondrial function secondary to changes in physiologic parameters and oxygen availability create a bioenergetic imbalance that may interfere with the balanced functioning of these pathways and thus contribute to conditions such as chronic pain, anhedonia, and mood disorders [43,44].

**Figure 2:**
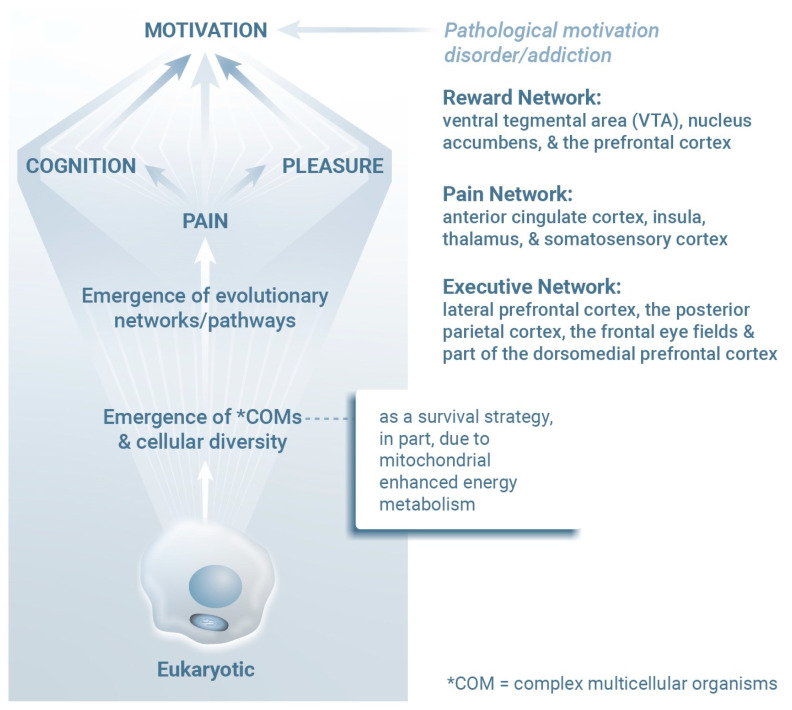
Mitochondria contribute to both energy production and oxygen detoxification processes that support the evolution of diverse eukaryotic cells as well as complex multicellular organisms. Energy availability, a critical factor leading to the evolution of complex life forms [56,57], most likely facilitated the development of interconnected neural networks that shape behaviors and responses essential for survival, including pleasure, pain perception, and cognition. These networks include the Reward Network, a CNS pathway that includes the ventral tegmental area, nucleus accumbens, and prefrontal cortex), the Pain Network (including the anterior cingulate cortex, insula, thalamus, and somatosensory cortex), and the Executive Network (including the lateral prefrontal cortex, posterior parietal cortex, frontal eye fields, and the dorsomedial prefrontal cortex). Interactions between these networks provide multicellular organisms with functional advantages when novel adaptive behaviors and survival strategies are needed.

The central mediators of neurocognitive responses include the ventral tegmental area (VTA), nucleus accumbens (NAc), and the prefrontal cortex (PFC); others include the anterior cingulate cortex (ACC), insula, thalamus, somatosensory cortex, lateral prefrontal cortex (LPFC), posterior parietal cortex (PPC), frontal eye fields, posterior cingulate cortex (PCC), and the dorsomedial prefrontal cortex (dmPFC) (Figure 2). As a group, these central structures exhibit significant overlap and interdependence with respect to their roles in supporting cognition, pain, pleasure, and motivation [45,46,47,48,49], relying on integrated neural networks in which each region contributes specialized tasks to produce coherent experiences and behaviors. For example, the mesolimbic pathway [50] (which includes the VTA and the NAc) links pleasure and motivation, with dopamine release in the NAc modulated by cognitive input from the PFC, i.e., mesocortical system. The salience network [51] (including the ACC, insula, and thalamus) balances pain, emotional significance, and motivation (Figure 2). Additionally, the default mode (the dmPFC and PCC) and executive control networks (including the PPC and LPFC) collaborate to support goal maintenance and adaptive responses. For example, a motivational response to a challenge begins with sensory inputs processed in the thalamus and somatosensory cortex, evaluated by the insula and ACC, reinforced by reward signals from the VTA and NAc, and culminating in goal-directed planning in the PFC [52]. Importantly, pain perception and emotional valence are processed, in part, by interoceptive signaling [53]. In this pathway, the ACC has a role in generating the emotional and evaluative components of pain [54]. The somatosensory cortex is responsible for detecting and localizing pain stimuli, and the thalamus relays these sensory inputs to higher-order regions of the brain. Meanwhile, the PFC modulates the cognitive appraisal of pain, including coping strategies [55].

A sustained oxygen supply is essential for cellular and systemic health. Hypoxic events can disrupt this balance, triggering inflammatory cascades and potentially leading to neurodegeneration and other systemic pathologies [1]. Perturbations in one area, such as mitochondrial dysfunction or tissue-specific inflammation, can have cascading effects on other interconnected systems, particularly the nervous system. Furthermore, prolonged or unresolved hypoxic events can activate and amplify pro-inflammatory pathways, leading to biological senescence (i.e., aging at the cellular level), energy deficits, and systemic decline.

Neural circuits have significantly different metabolic demands, and this variability contributes to their differential sensitivity to oxygen deprivation. Executive networks, such as the prefrontal cortex, and reward-related areas such as the ventral striatum and midbrain dopamine system, are among the most energy-demanding circuits in the brain due to their high synaptic activity, dense connectivity, and reliance on oxidative metabolism [31,58]. The prefrontal cortex, in particular, demonstrates elevated glucose utilization and oxidative phosphorylation rates (i.e., functions tightly coupled to mitochondrial ATP production) when engaged in tasks that require sustained attention, working memory, and decision making [59]. Similarly, midbrain dopamine neurons involved in reward signaling exhibit high basal firing rates and are especially vulnerable to hypoxic and oxidative stress due to their large axonal arborization and pacemaking activity [31,60]. The results of several experimental studies revealed that these regions exhibit early functional impairment during hypoxia or ischemia; by contrast, lower-order sensory areas exhibit greater resilience [61,62]. Thus, the selective vulnerability of executive and reward circuits to hypoxic conditions appears to be closely linked to their intrinsic energy demands and limited metabolic reserve. Further comparative metabolic studies will be needed to provide detailed support for the apparent differential oxygen sensitivity.

**Figure 3:**
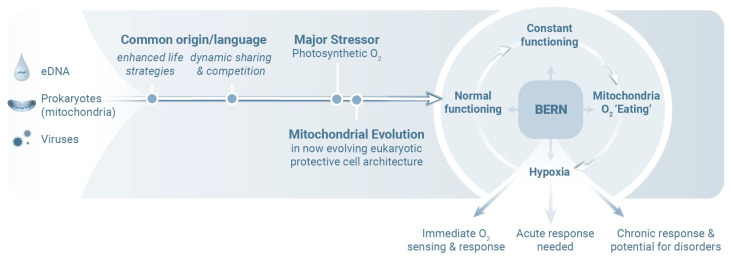
Mitochondria are integral protective and energy-generating cellular components that function continuously and consume oxygen (O_2_), thereby also reducing oxygen-related toxicity (i.e., oxidative stress); their capacity to sense and respond to O_2_ is a fundamental feature of eukaryotic life. Chronic disorders arise, at least in part, when mitochondria become incapable of efficient oxygen metabolism. HIF-1-mediated hypoxia is a critical trigger of inflammation, neurodegeneration, and mitochondrial dysfunction [69,70]. These broad-reaching observations reinforce the essential role of oxygen homeostasis in maintaining health and well-being. The Behavior, Exercise, Relaxation, and Nutrition (BERN) framework highlights several essential tools that may be used to optimize mitochondrial efficiency and resilience. Life is typically defined by an organism’s ability to grow, reproduce, respond to stimuli, metabolize, and maintain internal stability. Both viruses and mitochondria display many of these traits, extending the definition of what might be considered truly alive. Viruses can replicate under specific conditions, influence cellular metabolism, and evolve rapidly by adapting to their environments. Likewise, although they are now integrated within eukaryotic cells, mitochondria originated from free-living bacteria and still retain some independence, including the ability to function outside the cell. Both entities likely emerged from early life-like systems, for example, coacervates, which are droplets rich in genetic material (possibly environmental DNA) that facilitated rapid mutation and adaptation. While mitochondria are essential for energy production and oxygen regulation in cells, prolonged activity can lead to damaging byproducts, including ROSs, which are linked to aging and disease [19]. Further information on the theoretical framework of BERN as well as its scientific evidence and clinical mind–body medical applications, i.e., their empirical and practical validation, can be found in [63,71,72,73,74,75,76,77,78,79,80,81,82,83,84,85,86] and references within.

The Behavior, Exercise, Relaxation, and Nutrition (BERN) mind–body operational framework for healthy living [63] is based on many of these aforementioned principles (Figure 3) [64,65]. Different BERN techniques designed to promote health behavior change may act via shared autoregulatory CNS reward and motivation circuits [66]. Mind–body processes emphasize the links between health, energy regulation, and neural integrity. As noted above, the neural and immune systems are linked to one another and share both anatomical and biochemical feedback pathways [67,68]. This communication ensures coordinated responses to environmental and physiological changes and highlights mechanisms that include integrated defense, repair mechanisms, and energy sharing. Taken together, these findings lead us to conclude that supporting appropriate mitochondrial function, maintaining oxygen homeostasis, and mitigating inflammation are all strategies that might be used to sustain mind–body health and prevent pathophysiological outcomes.

The BERN process recognizes the need to balance energy, oxygen, and communication within the body and emphasizes the importance of maintaining equilibrium as a means to support long-term health and resilience. The BERN framework is based on mind–body practices that focus on cellular and molecular processes that have evolved for over two billion years to promote health and longevity.

Mitochondrial function plays a central role in sustaining mind–body health by integrating metabolic energy production with cellular signaling pathways that regulate stress responses, inflammation, and oxygen sensing. As the primary site of oxidative phosphorylation, mitochondria are responsible for generating the ATP needed to support neuronal activity, hormonal regulation, and immune competence, processes that are fundamental to both cognitive and physiological well-being [87]. Mitochondria also serve as oxygen sensors by helping to maintain oxygen homeostasis by regulating ROSs and stabilizing hypoxia-inducible factors, which coordinate adaptive responses to stress and low oxygen availability [88]. Disruptions in mitochondrial respiration or redox signaling can impair one or more of these compensatory mechanisms. This can lead to chronic inflammation, oxidative stress, and metabolic dysfunction, all hallmarks of many diverse pathophysiological states, including depression, cardiovascular disease, and neurodegeneration [89]. Anti-inflammatory signaling mediated through mitochondrial pathways, including activation of sirtuin 3, peroxisome proliferator-activated receptor gamma coactivator 1-alpha, and mitophagy, contributes to the resolution of systemic inflammation and promotes resilience in both brain and body systems [90].

## 3. Psychiatric Ramifications

Mitochondria and their contributions to oxygen/energy processing may have a profound albeit incompletely understood role in the pathogenesis of neuropsychiatric disorders [90,91]. Among the many examples, Andreazza and colleagues [92] reported increases in protein oxidation and nitration in association with diminished mitochondrial complex I activity in a series of postmortem samples of frontal cortex from patients diagnosed with bipolar disorder. Scaini and colleagues [93] reported differential gene expression, suggesting mitochondrial fragmentation in leukocytes from patients with major depressive disorder; more recently, Ye and colleagues identified circulating mitochondrial DNA as a biomarker for this disorder [94]. In 2022, Roberts [95] published a comprehensive review on mitochondrial dysfunction in schizophrenia, highlighting deficits in mitochondrial gene expression, electron transport chain components, as well as diminished numbers of mitochondria in axon terminals and oligodendrocytes throughout the cerebral cortex.

Mitochondria are far more than cellular powerhouses; they are sensitive, adaptive hubs that facilitate internal communication and cellular responses to their environment. Their ability to sense changes in oxygen levels, energy demand, calcium levels, and stress allows them to adjust energy production in real time, support survival, and initiate protective responses when needed [96]. These properties, collectively defined as mitochondrial sensitivity, are especially vital in energy-demanding tissues such as the brain, where the precision of neuronal signaling depends on the availability of a constant, well-regulated energy supply. Here, mitochondria provide energy to processes such as neurotransmitter release and network synchronization, which are essential for memory, attention, and perception [31,58]. The link between energy production and communication illustrates the concept of energy-based information exchange, which is the idea that living systems use energy not just to function, but to carry and process information. When mitochondrial function is disrupted, energy–information coupling breaks down, contributing to cognitive decline and disease [97]. Thus, mitochondria play a unique and dynamic role in both sensing internal conditions and enabling cells to communicate through energy processing.

In addition to our current understanding of the outcomes of mitochondrial dysfunction, the predictive processing hypothesis offers a novel framework for understanding psychiatric diseases such as schizophrenia [98]. The predictive processing hypothesis suggests that the disturbed neuronal communication characteristic of this disorder arises from perturbations in energy-based information exchange. Energy-based information exchange, which focuses primarily on learned stimulus thresholds (i.e., experience), operates in a top-down manner with external sensory inputs undergoing constant evaluation. In this process, external sensory signals are constantly adjusted to match the expectations and ultimately transferred together with unresolved noise (i.e., prediction errors) to deeper neural structures in which information is processed based on one’s recognition of the stimulus (i.e., signal) and its similarity to earlier experiences. Initially unmatching stimuli (i.e., signals that are not recognized) will cause disruptions in communication and an increase in free energy (or entropy). The excess free energy generated by communication disruptions must be reduced if the system is to regain equilibrium and restore healthy cognitive functioning. Mitochondrial dysfunction may disrupt the pathways involved in reducing free energy, which may ultimately lead to a mismatch of information processing. Here, we are referring to the “free-energy principle” as defined by neuroscientists, meaning the theory that the brain attempts to reduce variational free energy as a way to reduce uncertainty and maintain a stable state. First introduced by Karl Friston [99], this principle proposes that the brain maintains homeostasis by reducing variational free energy, which is a proxy for surprise or prediction error. Based on this principle, neural circuits act as predictive engines, forming internal models that anticipate sensory inputs and update them through prediction error signals, thereby conserving energy and optimizing function [100].

The efficient coding hypothesis [101] provides strong support for this concept. This hypothesis suggests that sensory neurons encode stimuli with minimal redundancy. For example, retinal and cortical neurons respond more strongly to novel stimuli than to predictable ones, thereby reducing unnecessary spikes and metabolic load [58]. This is extended by predictive coding models, such as those proposed by Rao and Ballard [102], that describe cortical hierarchies that transmit predictions. Only mismatched signals (prediction error) are transmitted upward, a process that facilitates focused and energy-efficient signaling.

Similarly, results from empirical studies revealed that neurons in the visual cortex increase their activity when confronted with omissions or other unexpected phenomena. For example, Keller et al. [103] reported that mice trained in virtual environments displayed anticipatory neural activity in V1 and heightened responses when expected stimuli were omitted. These effects are mediated by feedback from higher-order areas, for example, the anterior cingulate cortex, emphasizing top-down modulation of predictive processing.

Sensorimotor systems are also based on energy-based predictive exchange. Forward models predict sensory consequences during movement, which dampen the responses to self-generated stimuli via a mechanism known as sensory attenuation [104]. This mechanism conserves energy by avoiding redundant processing. In the cerebellum, climbing fibers encode motor prediction errors and can thus drive adaptation to minimize future mismatch [105].

Reward-related circuits also illustrate this principle. Dopaminergic neurons encode reward prediction errors: they fire when outcomes deviate from expectations, thereby facilitating learning [106]. These phasic signals allocate neural and metabolic resources to stimuli and events that are unexpected, and thus informative. Limbic areas (e.g., the orbitofrontal cortex and anterior cingulate) also generate predictions regarding emotional and interoceptive states and thus contribute to adaptive allostasis [107].

The term “mismatch” refers to the match/mismatch hypothesis, which posits that individuals’ responses and behaviors are influenced by the degree of match or mismatch between their current experiences and their expectations based on past experiences [99,108]. Ongoing stress may further exacerbate these problems, thereby creating false associations underlying symptoms (e.g., hallucinations) [98]. The principles of the energy-mismatch model go beyond the limitations of traditional views and provide an approach that may not depend directly or completely on specific neurotransmitters [98]. For example, the energy-match model also facilitates cognitive in silico modeling based on neuronal energy transfer and exchange, rather than remaining limited to the variability of neurotransmitter profiles. At its core, the energy-mismatch hypothesis is a straightforward and transformative concept that considers brain function by analogy to a computer operation.

Recent work reinforces these principles through computational modeling and neuroimaging. Bakhtiari [109] showed that artificial neural networks trained under energy constraints spontaneously develop predictive coding architectures. Meta-analyses performed by Ficco et al. [110] identified a common prediction-error network across sensory modalities, including the insula and frontal regions. Taken together, these findings support the conclusion that neural systems can reduce free energy and optimize energetic efficiency through predictive processing across sensory, motor, and emotional domains.

## 4. Evolutionary Efficiency

As described above, specific regions in the CNS perform distinct roles but are also capable of dynamic interactions that enable the brain to synthesize diverse neural activities into cohesive human behavior [111]. This shared anatomical and physiological framework features an evolutionarily economical strategy for survival in which multitasking centers efficiently mediate complex behaviors. However, ongoing rapid integration of interdependent CNS processes depends on immediate energy availability, primarily through mitochondrial activity [7]. Acute and chronic hypoxia provide critical input to ensure that neural networks respond appropriately to a given stress [112,113,114]. Thus, it is perhaps not surprising that mitochondrial sensitivity and dysfunctional responses to hypoxia might have an immediate impact on cognitive behavior. Tapping into this sensitivity also manifests itself in various disorders as well as in response to pathological agents, further highlighting the significance of oxygen for effective signal integration.

## 5. Conclusions

In summary, mitochondria represent a pivotal evolutionary innovation in which ancestral bacteria were transformed into energy powerhouses that provide essential support for eukaryotic complexity and maintain systemic health. Mitochondria are also critical features of bidirectional neural–immune communication and adaptive responses that rely on oxygen-driven ATP production. The interplay between hypoxia-induced mitochondrial dysfunction and dysregulated energy metabolism has been identified as a critical trigger of inflammation, which can lead to systemic decline, neurodegeneration [115], and dysfunctional neurocognition. The BERN framework focuses on behavior, exercise, relaxation, and nutrition as a means to optimize mitochondrial efficiency and physiological resilience. Collectively, these insights underscore the pivotal role of mitochondria in sustaining health and longevity and offer a fresh perspective on our understanding of mental health and neuropsychiatric disease.

## Abbreviations

The following abbreviations are used in this manuscript:

ADP	adenosine diphosphate
ATP	adenosine triphosphate
BERN	Behavior, Exercise, Relaxation, and Nutrition
DNA	deoxyribonucleic acid
eDNARNA	environmental DNAribonucleic acid
MROs	mitochondrion-related organelles
CNS	central nervous system
VTA	ventral tegmental area
NAc	nucleus accumbens
PFC	prefrontal cortex
ACC	anterior cingulate cortex
LPFC	lateral prefrontal cortex
PCC	posterior cingulate cortex
PPC	posterior parietal cortex
dmPFC	dorsomedial prefrontal cortex
HIF	hypoxia-inducible factor
ROSs	reactive oxygen species
